# The association between different types of childhood trauma and orthorexia nervosa: an observational study

**DOI:** 10.3389/fpsyg.2025.1665790

**Published:** 2025-11-05

**Authors:** Francesca Pesavento, Alessandro Alberto Rossi

**Affiliations:** ^1^Department of Philosophy, Sociology, Education, and Applied Psychology, Section of Applied Psychology, University of Padova, Padova, Italy; ^2^Center for Intervention and Research on Family Studies – CIRF – Department of Philosophy, Sociology, Education, and Applied Psychology, Section of Applied Psychology, University of Padova, Padova, Italy

**Keywords:** orthorexia nervosa, adverse childhood experiences, childhood trauma, disordered eating behaviors, emotional abuse and neglect, eating behaviors

## Abstract

**Objective:**

Orthorexia Nervosa (ON) is characterized by the pursuit of perfect eating, leading to severe physical, psychological, and social problems. Although not yet formally recognized as an official diagnosis, ON shares etiological factors with traditional eating disorders, such as adverse childhood experiences (CT). This study explores the association between different CT types and ON, a relationship scarcely investigated in the literature.

**Methods:**

A sample of 175 participants (age 18–74 years, M = 44.10; SD = 12.85) completed the Italian Düsseldorf Orthorexia Scale (I-DOS) and the Childhood Trauma Questionnaire (CTQ-SF), assessing Physical Abuse (PA), Emotional Abuse (EA), Sexual Abuse (SA), Physical Neglect (PN), and Emotional Neglect (EN). A backward linear regression analysis investigated the CT-ON association.

**Results:**

The regression model explained 7.8% of variance in ON. EA emerged as the strongest predictor. SA showed limited relevance. The negative association with PA likely reflects statistical suppression effects and should be interpreted with caution. PN and EN showed positive but non-significant associations with ON.

**Conclusion:**

These findings support the role of CT, particularly EA, in ON development. EA represents the most clinically relevant trauma type, suggesting therapeutic interventions should prioritize addressing emotional abuse. However, the modest variance explained indicates that future studies should investigate additional psychological factors (e.g., perfectionism, cognitive restraint, attachment) to gain comprehensive understanding of ON etiology. This exploratory study provides foundational knowledge for future research on CT’s role in ON.

## Introduction

In recent years, there has been growing attention to rigid self-imposed diets characterized by the pursuit of food that – often from a subjective perspective – is considered “healthy.” Whether restrictive or simply selective, these eating habits appear to be associated with an excessive concern for health and food quality: this is the case of orthorexia nervosa (ON) (Donini et al., 2022). Literature highlighted that ON represents an emerging disordered eating behavior that only partially overlaps with traditional eating disorders (EDs) such as anorexia nervosa and bulimia nervosa. Among their points of convergence, ON appears to share certain etiological factors such as the presence of emotional/relational trauma and adverse childhood experiences.

### Childhood trauma and eating disorders

Relational childhood traumatic (CT) experiences appears to be a widespread issue, often characterized by abusive and/or neglectful relationships with caregivers during infancy (Bernstein et al., 2003). CT is associated with impaired emotion regulation and self-development (Schimmenti, 2014), leading to psychological vulnerability and various effects in adulthood (Van Veen et al., 2013; Huh et al., 2014; Schimmenti, 2014; Innamorati et al., 2016). In most cases, CT is perpetrated by adults (Bernstein et al., 2003), parents, relatives, teachers, or strangers. CT can occur in many ways and a differentiation of CT has been proposed (Bernstein et al., 2003): sexual abuse (SA), physical abuse (PA), emotional abuse (EA), physical neglect (PN), and emotional neglect (EN). Experiences of PA involve the enactment of physically violent behaviors by caregivers toward the child, whether in the context of disciplinary punishment or not, often leading to physical injuries such as bruises and, in some cases, requiring medical attention (Bernstein et al., 2003). SA occurs when an adult engages a minor in sexual activities, regardless of whether physical force is used or the child appears to comply, as minors cannot provide valid consent to such behaviors (Bernstein et al., 2003). EA is understood as a set of verbal assaults and behaviors aimed at humiliating or damaging the victim’s sense of self-worth, for example by addressing the child with offensive names, making them feel hated or unwanted (Bernstein et al., 2003). Lastly, PN and EN are defined as the caregivers’ inability to meet the child’s physical or emotional/psychological needs, preventing them from experiencing safety, belonging, and love. PN occurs when the child is not provided with access to clean clothing, sufficient food, or adequate parental care, sometimes in the context of substance abuse (Bernstein et al., 2003). Instead, EN limits the child’s perception of living in a cohesive and supportive family and diminishes the sense of being loved and valued (Bernstein et al., 2003; Mannarini et al., 2018; Panzeri et al., 2022).

Childhood traumatic experiences tend to elicit adverse psychopathological conditions across the lifespan (Schimmenti and Caretti, 2016a; Midolo et al., 2020; Santoro et al., 2025b). The negative impact of CT can manifest during childhood and adolescence, but often extends into adulthood (Norman et al., 2012; Hailes et al., 2019). Specifically, CT are associated with increased risk of depressive disorders, substance abuse, and suicidal behaviors in adulthood (Norman et al., 2012; Hailes et al., 2019; Li et al., 2016), as well as difficulties in managing one’s internal states (Schimmenti, 2018).

Among these adverse consequences, the relationship between CT and EDs is well-documented (Caslini et al., 2016; Barakat et al., 2023), ED’s symptoms stem from painful internal states (Santoro et al., 2025a), that are not integrated into conscious awareness (Schimmenti, 2014; Schimmenti and Caretti, 2016b). Overall, the literature identifies SA (Afifi et al., 2017) and EA (Monteleone et al., 2019) as the two main predictors of EDs whether PA, EN, and PN appear to be weaker and more inconsistent associated with EDs (Rayworth et al., 2004; Norman et al., 2012; Pignatelli et al., 2017; Carvalho Silva et al., 2024; Amiri and Sabzehparvar, 2025). On one hand, SA is associated with body dissatisfaction (Caslini et al., 2016), representing a transdiagnostic factor for bulimia nervosa (BN), binge eating disorder (BED), and anorexia nervosa (AN), and is linked to restrictive eating behaviors, binge eating, and purging (Connors, 2001; Rayworth et al., 2004; Caslini et al., 2016). On the other hand, EA is believed to serve as a bridge – a mediator – between other childhood traumatic experiences and the development of ED-related symptoms (Monteleone et al., 2019). Indeed, EA undermines one’s sense of efficacy, impairing emotional regulation, and disrupting coping strategies (Caslini et al., 2016; Monteleone et al., 2019). Furthermore, EA has been associated with AN (Caslini et al., 2016; Monteleone et al., 2019) and linked to issues with self-esteem and self-perception (Hymowitz et al., 2017).

### Orthorexia nervosa

Despite until now most of research attention has focused on AN, BN and BED, there are more subtle and less studied eating-related conditions that may also be associated with CT (Rossi et al., 2025a). Among these emerging conditions, ON represents a particularly relevant case. ON is a condition characterized by an intense concern with maintaining and improving one’s health through the obsessive pursuit of a putative perfectly healthy diet (Donini et al., 2022), a tendency often reinforced by the widespread dissemination of – frequently inaccurate – information about “proper nutrition (Bratman, 2017; Horovitz and Argyrides, 2023; Rzeszutek et al., 2024). Although not yet recognized as an official diagnosis, ON is often associated with EDs (Donini et al., 2022; Zagaria et al., 2022; Rossi et al., 2024) with estimates of its prevalence in the general population ranging from 1 to 8% (Zagaria et al., 2022).

The association between ON and CT appears plausible due to ON’s features, which overlap with those found in trauma-related EDs. Indeed, the rigidity toward self-imposed rules in meal preparation (Donini et al., 2022) and the increasing rigidity regarding “allowed” foods (Barthels et al., 2015) may serve to enhance a sense of safety and self-esteem (Hymowitz et al., 2017), often compromised by CT (Cheshire et al., 2020). Food selection, based on cognitive evaluation rather than appetite (Barthels et al., 2015), reflects the need for control – a core trait in ON. In this sense, ON may act as a coping strategy (Zagaria et al., 2024), aimed at countering feelings of inefficacy (Brytek-Matera et al., 2020), managing negative emotions – often impaired by CT (Rzeszutek et al., 2024) – and preserving one’s health (Barthels et al., 2015); despite this eating pattern can lead to negative physiological, individual, and social outcomes (Bratman, 2017; Donini et al., 2022; Horovitz and Argyrides, 2023). Moreover, in ON the concern with food purity (Bratman, 2017; Donini et al., 2022; Rossi et al., 2024), may reflect underlying feelings of shame and impurity (Bratman, 2017), which have been linked to physical and emotional CT (Cheshire et al., 2020). Finally, weight loss is observed in individuals with ON (Donini et al., 2022), While this is typically regarded as an unintended consequence not pursued for aesthetic reasons (Donini et al., 2022), there also appears to be an association between ON and a negative perception and experience of one’s own body (Barthels et al., 2015; Brytek-Matera et al., 2020). Since experiencing discomfort or discontent with one’s physical appearance is strongly associated with SA (Caslini et al., 2016; Cheshire et al., 2020), these experiences should be further explored to enhance clinical interventions in ON.

## Aims

In light of this background, a strong link between EA and ON is plausible, as EA is a cross-cutting risk factor in EDs (Afifi et al., 2017; Monteleone et al., 2019) and is associated with AN (Caslini et al., 2016; Monteleone et al., 2019), which is often compared to ON (Atchison and Zickgraf, 2022). SA may be less relevant to ON, given conflicting findings on body image (Donini et al., 2022). PA, EN, and PN are thought to marginally contribute to ON (Rayworth et al., 2004; Amiri and Sabzehparvar, 2025).

However, to date, there appear to be no scientific studies exploring the association between specific childhood traumatic experiences and the presence of ON symptoms.

Therefore, the present study aims to investigate the relationship between major childhood traumatic components and ON. Given the limited research on the relationship between CT and ON, the present study adopts a focused exploratory approach, examining only CT types as predictors. This strategy aims to establish baseline knowledge about CT’s role in ON before examining more complex multivariate models in future research.

## Materials and methods

### Procedure

Data were collected online through a battery of self-report questionnaires administered using the Qualtrics software. Recruitment was conducted via major social media platforms (e.g., Facebook), targeting the general population using convenience snowball sampling. Inclusion criteria required participants to understand the Italian language, be at least 18 years old, and have provided informed consent. Moreover, any questionnaires with incomplete responses were excluded – thus, the dataset contained no missing data.

### Participants

The final sample consisted of 175 individuals, of whom 8% were male (*n* = 14) and 92% female (*n* = 161). Participants’ ages ranged from 18 to 74 years (M = 44.10; SD = 12.85), and body mass index (BMI) ranged from 14.34 to 47.75 (M = 25.70; SD = 12.85) (Table 1).

**Table 1:** Descriptive statistics of the sample.

Variables	Descriptive statistics	
Age (M, SD)	44.100	12.850
BMI (M, SD)	25.703	5.816
Biological sex (*n*, %)		
Male	14	8%
Female	161	92%
Marital status (*n*, %)		
Single – not in a relationship	26	14.90%
Single – in a relationship	50	28.60%
Married	83	47.40%
Separated/Divorced	12	6.90%
Widowed	4	2.30%
Education level (*n*, %)		
Lower secondary school	21	12.00%
Upper secondary school	93	53.10%
University degree	46	26.30%
Master’s/PhD	15	8.60%
Occupation (*n*, %)		
Student	12	6.90%
Full-time employee	70	40.00%
Part-time employee	31	17.70%
Self-employed	32	18.30%
Domestic helper/housekeeper	9	5.10%
Unemployed	8	4.60%
Retired	13	7.40%

M = mean, SD = standard deviation, *n* = number of participants, % = percentage.

### Instruments

#### Italian Düsseldorf orthorexia scale (I-DOS)

The Düsseldorf Orthorexia Scale (Barthels et al., 2015) is a 10-item self-report questionnaire designed to assess orthorexic attitudes and behaviors with a 4-point Likert scale (Cerolini et al., 2022). A score up to 25 suggests a risk of ON, while a score ≥ 30 indicates the presence of ON. The I-DOS also shows correlations with general ED psychopathology. In the present sample, the scale demonstrated good internal consistency (*α* = 0.861).

#### Childhood trauma questionnaire-short form (CTQ-SF)

The Childhood Trauma Questionnaire-Short Form (Bernstein et al., 2003) is a self-report instrument that assesses childhood traumatic experiences. It consists of 28 items rated on a 5-point Likert scale. The Italian version retains the original structure and includes five clinical subscales corresponding to the main types of childhood trauma: PA, SA, EA, EN, and PN (Sacchi et al., 2018). In the current study, the internal consistency for each subscale was as follows: EN (α = 0.897), EA (α = 0.825), PA (α = 0.764), SA (α = 0.707), and PN (α = 0.567).

### Statistical analyses

Statistical analyses were performed using JASP. Bivariate correlations were computed, examined to assess the strength of associations between different types of CT and ON, as well as skewness, and kurtosis. Following Cohen’s (1988) guidelines, correlations were interpreted as small for |0.10| ≤ *r* < |0.30|, medium for |0.30| < *r* < |0.50|, and large for *r* ≥ |0.50|. However, particular attention was paid to the presence of bivariate correlations greater than |0.85|, as they could indicate potential problems in the construct definition (Rönkkö and Cho, 2022; Panzeri et al., 2024b).

Finally, a backward linear regression analysis was conducted, using the total score of the I-DOS (ON) as the dependent variable and the CTQ-SF subscales (EA, PA, SA, EN, and PN) as independent variables. The regression model was deliberately designed to focus exclusively on childhood trauma types as predictors of ON, without including other variables commonly associated with ON (e.g., perfectionism, cognitive restraint, attachment styles, obsessive traits, body image concerns). This focused approach allows for a clear examination of the specific contribution of CT to ON symptomatology, providing foundational knowledge for future, more comprehensive models.

Unstandardized regression coefficients (*β*) were reported into the text assessing the contribution of each independent variable on ON attitudes and behaviors across models. The adjusted *R^2^* was evaluated across the different models – indicating the percentage of variance in ON attitudes and behaviors explained by the predictors, from Step 1 to Step 4. The backward method was chosen over the forward method because it is less prone to Type II errors. Starting with all independent variables reduces the risk of excluding potentially influential predictors due to suppression effects. This type of linear regression is effective for deriving the most parsimonious model: independent variables were entered individually starting at Step 1, and in Steps 2, 3, and 4, were progressively removed if they were not statistically significant. Additionally, the backward model allows for a re-evaluation of the remaining predictors at each step.

## Results

### Preliminary analysis

Several preliminary analyses were carried out — such as the assessment of variance inflation factor (VIF) and tolerance — to verify that the assumptions of the regression model were met. No violations of the assumptions were found, and therefore the model was executed.

*Correlation analysis.*

The correlation analysis revealed weak associations between ON and CT experiences (Table 2). EA showed the highest correlation among all independent variables (*r* = 0.256), but still indicated only a small effect on ON. Regarding other correlations with ON, PA and EN showed no statistically significant effects. Both PN and SA had small correlations with ON (*r* = 0.175, *r* = 0.158). When examining correlations among the different CT types, EA was the only independent variable statistically significantly correlated with all the other CT, showing a strong association with PN (*r* = 0.515), EN (*r* = 0.732), and PA (*r* = 0.541), and a smaller effect with SA (*r* = 0.245).

**Table 2:** Descriptive statistics and Pearson’s bivariate correlations.

		Descriptive statistics				Correlations				
		M	SD	Skewness	Kurtosis	1	2	3	4	5
1	ON	17.143	5.394	0.902	0.819	-				
2	PN	6.743	2.121	1.564	3.293	0.175^*^	-			
3	EA	8.520	3.832	1.059	0.371	0.256^**^	0.515^**^	-		
4	EN	10.829	4.465	0.695	−0.263	0.126	0.519^**^	0.732^**^	-	
5	PA	5.869	1.903	2.530	5.093	0.009	0.440^**^	0. 541^**^	0.491^**^	-
6	SA	5.920	2.987	4.283	19.741	0.158^*^	0.069	0.245^**^	0.180^*^	0.057

* = *p* < 0.05 (2-tailed), ** = *p* < 0.001 (2-tailed). M, mean; SD, standard deviation; ON, Orthorexia Nervosa; PN, Physical Neglect; EA, Emotional Abuse; EN, Emotional Neglect; PA, Physical Abuse; SA, Sexual Abuse.

### Regression analysis

The backward linear regression (Table 3) provided four steps. Step 1 explained 8.6% of the variance in ON (adjusted *R^2^* = 0.086, *p* = 0.001), including all five CT types: PN (*β* = 0.324, 95%CI[−0.104, 0.752]), EA (*β* = 0.511, 95%CI[0.190, 0.832]), SA (*β* = 0.170, 95%CI[−0.098, 0.439]), PA (*β* = −0.523, 95%CI[−1.022, −0.023]), EN (*β* = −0.164, 95%CI[−0.427, 0.100]).

**Table 3:** Results of backward regression analysis: childhood trauma on orthorexia nervosa symptoms (*n* = 175).

	β*	*β* SE	95% CI	*t*	*p-*value	*F*	Adj *R^2^*	VIF	*T*
Step 1					0.001	4.274	0.086		
PN	0.133	0.324 (0.217)	[−0.104, 0.752]	1.493	0.137			1.507	0.664
EA	0.363	0.511 (0.163)	[0.190, 0.832]	3.140	0.002			2.541	0.393
SA	0.094	0.170 (0.136)	[−0.098, 0.439]	1.255	0.211			1.077	0.929
PA	−0.184	−0.523 (0.253)	[−1,022, −0.023]	−2.064	0.041			1.519	0.658
EN	−0.135	−0.164 (0.133)	[−0.427, 0.100]	−1.226	0.222			2.322	0.431
Step 2					0.001	4.952	0.083		
PN	0.109	0.267 (0.212)	[−0.152, 0.685]	1.257	0.211			1.437	0.696
EA	0.284	0.399 (0.135)	[0.133, 0.666]	2.956	0.004			1.748	0.572
SA	0.092	0.166 (0.136)	[−0.103, 0.434]	1.219	0.225			1.076	0.930
PA	−0.198	−0.560 (0.252)	[−1.057, −0.063]	−2.225	0.027			1.497	0.668
Step 3					0.001	6.090	0.081		
PN	0.104	0.254 (0.212)	[−0.165, 0.673]	1.196	0.233			1.433	0.698
EA	0.314	0.441 (0.131)	[0.183, 0.699]	3.375	0.001			1.634	0.612
PA	−0.206	−0.584 (0.251)	[−1.081, −0.088]	−2.326	0.021			1.487	0.672
Step 4					<0.001	8.399	0.078		
EA	0.354	0.499 (0.122)	[0.258, 0.739]	4.097	<0.001			1.413	0.708
PA	−0.182	−0.517 (0.245)	[−1.001, −0.033]	−2.109	0.036			1.413	0.708

β* = standardized regression coefficient; *β* = unstandardized coefficient; SE = standard error; 95% CI = 95% confidence interval; *t* = t-value; *p* = significance level; *F* = model F-test; Adj *R^2^* = adjusted coefficient of determination; VIF = variance inflation factor; *T* = tolerance. Predictors included are: PN = Physical Neglect, EA = Emotional Abuse, SA = Sexual Abuse, PA = Physical Abuse, EN = Emotional Neglect.

Between Step 1 and Step 4, the backward regression progressively eliminated non-significant predictors. At Step 2, EN was removed (the weakest predictor with the lowest t-value), resulting in minimal impact on model fit. At Step 3, SA was excluded due to its non-significant contribution. Finally, at Step 4, PN was also removed. Across this process, the adjusted *R^2^* dropped by less than 1% (from 0.086 to 0.078), which is reasonably considered negligible. This stepwise elimination resulted in a more parsimonious model while maintaining explanatory power (see Table 3).

The final model – Step 4 – Included only EA and PA as predictors of ON, explaining 7.8% of the variance (adjusted *R^2^* = 0.078, *p* < 0.001): EA (*β* = 0.499, 95%CI[0.258, 0.739]), PA (*β* = −0.517, 95%CI[−1.001, −0.033]). Consequently, the final model at Step 4 can be considered more parsimonious and robust. Moreover, the negative *β* value observed for PA, suggests an inverse relationship with the dependent variable ON.

## Discussion

This study aimed to explore the impact of various CT experiences on orthorexic attitudes and behaviors, a topic still under-investigated in the literature, not yet included in diagnostic manuals, and associated with dysfunctional eating behaviors. Understanding this association can enhance knowledge in the field and help guide clinical interventions. ON appears to be characterized by an excessive concern for one’s health, which individuals attempt to maintain and improve through what they perceive as a perfectly healthy diet (Donini et al., 2022). In this condition is likely to be present a marked inflexibility and increasing rigidity toward self-imposed dietary rules (Barthels et al., 2015; Moroze et al., 2015; Bratman, 2017; Donini et al., 2022; Rossi et al., 2025a). However, such behaviors often harm individual well-being, and social (Bratman, 2017; Donini et al., 2022; Horovitz and Argyrides, 2023) as well as physiological functioning (Donini et al., 2022).

Although ON is not yet formally recognized as a clinical diagnosis, growing evidence associates it with the spectrum of EDs (Donini et al., 2022; Zagaria et al., 2022; Rossi et al., 2024). For these reasons, it was hypothesized that ON, and EDs may share several risk factors, including CT (Caslini et al., 2016; Barakat et al., 2023) which is linked to a wide range of adult psychopathological conditions (Schimmenti and Caretti, 2016b). Literature has identified five main types of CT: PA, SA, EA, PN, and EN (Bernstein et al., 2003), this categorization would allow for a more nuanced understanding of trauma’s impact and enhances the precision of clinical intervention. However, ON remains poorly explored in terms of its prodromal factors including the various types of CT.

### Correlation analysis

Results from the present study showed that most types of CT were positively associated with ON attitudes and behaviors. These findings align with the literature regarding the impact of traumatic childhood experiences in individuals exhibiting symptoms typical of EDs (Caslini et al., 2016; Barakat et al., 2023). Among the independent variables, EA showed the strongest correlation with ON attitudes and behaviors, consistent with findings from several previous studies indicating that EA should be considered the strongest predictor of EDs (Afifi et al., 2017; Monteleone et al., 2019). This could be explained by the impact that EA experiences tend to have, generating a sense of ineffectiveness (Brytek-Matera et al., 2020), feelings of shame and impurity (Bratman, 2017), and damaged self-esteem (Hymowitz et al., 2017) – elements also present in ON (Monteleone et al., 2019; Cheshire et al., 2020; Barone et al., 2024). Furthermore, EA is associated with the development of poor emotional regulation strategies (Hatkevich et al., 2021), which also seems to occur in ON. In fact, within ON, difficulties in emotional regulation appear to trigger a need for control, expressed through orthorexic symptoms as a coping strategy (Vuillier et al., 2020). These findings align with theoretical perspectives suggesting that rigid behaviors in ON may serve compensatory functions, such as improving damaged self-esteem (Hymowitz et al., 2017), addressing feelings of shame and impurity (Bratman, 2017), and managing negative emotions (Pignatelli et al., 2017; Cheshire et al., 2020; Panzeri et al., 2024a; Rzeszutek et al., 2024; Rossi et al., 2025b). While the present study did not directly measure these mediating mechanisms, future research should investigate whether variables such as self-esteem, shame, and emotion regulation mediate the relationship between EA and ON symptoms.

Also, the results showed a non-statistically significant correlation between ON and EN, and a significant but small one with PN. This appears to support Hatkevich et al. (2021), who argue that EN affects emotion recognition more than regulation. Therefore, EN may be less associated with ineffective emotional regulation strategies (Hatkevich et al., 2021). Thus, emotion recognition should be less impaired in ON, since this issue seems to relate more to diagnoses such as BN, and BED (Hatkevich et al., 2021), which are less similar to ON compared to AN (Atchison and Zickgraf, 2022). As for PN, further studies may be needed to clarify its role in ON. However, its limited relevance in the etiology of EDs, as reported in the literature (Carvalho Silva et al., 2024), was reflected in the linear regression results, which will be discussed shortly.

The correlation between SA and ON attitudes and behaviors was small but statistically significant. This partially confirms findings from research on the influence of SA on ED symptoms (Afifi et al., 2017). Its weaker correlation with the dependent variable may be explained by the strong impact SA tends to have on body dissatisfaction (Caslini et al., 2016), which is not considered a central feature in ON (Donini et al., 2022). Finally, PA did not show a statistically significant correlation with ON attitudes and behaviors and had the smallest effect among the independent variables. This result contrasts with the literature, which generally supports the role of PA in EDs (Rayworth et al., 2004), despite ON not being formally included in it. However, the linear regression results revealed a different effect, which we will discuss shortly.

Regarding the correlations between the CTQ-SF subscales, the results obtained allowed for an assessment of whether some of them could mask the effects of others on the variance of the dependent variable in the linear regression model. It emerged that EA was the variable most strongly correlated with the others, showing a statistically significantly strong effect with PA, EN, PN, and a smaller effect with SA. This result has also been highlighted in previous studies (Mey et al., 2020) and appears to be consistent with the findings of Monteleone et al. (2019), who suggest that EA plays a mediating role between other types of CT and their psychopathological outcomes. In particular, the correlation between EA and PA is especially relevant to the linear regression results. The *r* effect size between these two variables is considered large (*r* = 0.541) and has been reported with similar values in other studies (Mey et al., 2020). This finding can be understood in light of literature suggesting that experiences of EA may increase susceptibility to instances of PA (Midolo et al., 2020).

### Regression model

Regarding the results of the linear regression, the final model at Step 4 identified EA and PA as the main predictors of ON attitudes and behaviors. In the case of EA, it was found to consistently maintain the highest regression coefficient among all variables across the four tested models. Thus, this result aligns with the established relevance of EA in eating disorder symptoms, as previously reported in the literature (Afifi et al., 2017; Monteleone et al., 2019), and with hypotheses about its impact on ineffectiveness (Brytek-Matera et al., 2020), self-esteem (Hymowitz et al., 2017), and emotional regulation (Hatkevich et al., 2021)—all of which are impaired in ON (Monteleone et al., 2019; Cheshire et al., 2020; Vuillier et al., 2020; Barone et al., 2024). The second predictor in the final regression model was PA, which showed a potentially inverse predictive effect on the dependent variable, as indicated by the negative sign. This result appears contradictory to the findings from the bivariate correlation, since the regression for PA should indicate the direct effect of the independent variable on ON. Therefore, not only did PA emerge as a predictor of ON attitudes and behaviors (despite the previously statistically non-significant correlation), but it did so with an effect opposite to that suggested by correlations. This outcome contradicts the literature, as PA is typically positively associated with the development of EDs (Rayworth et al., 2004). It can be hypothesized that the strong correlation between PA and EA, along with the association between EA and ON attitudes and behaviors, influenced the regression, making PA statistically significant in the model. Additionally, these associations may have produced a Simpson’s paradox, reversing the effect of PA. It is important to acknowledge that this statistical pattern substantially limits the interpretability of PA as a meaningful predictor of ON. The negative coefficient of PA in the final model, contradicting both the bivariate correlation and the established literature on EDs (Rayworth et al., 2004), strongly suggests that this result reflects a statistical artifact rather than a genuine protective effect. The high correlation between PA and EA (r = 0.541), combined with EA’s strong predictive role, likely created a suppression effect wherein PA’s regression coefficient was artificially reversed. Consequently, the final regression model should be interpreted as primarily identifying EA as the robust predictor of ON attitudes and behaviors, while the PA finding requires substantial caution and should not be over-interpreted. This phenomenon underscores the complexity of disentangling overlapping childhood trauma experiences and highlights a methodological challenge when examining highly intercorrelated predictors. In other words, it could be hypothesized that experiences of PA do not, in themselves, constitute a predictive factor of ON. Indeed, according to the literature, PA experiences frequently co-occur with EA (Brown et al., 2019): this suggests that physically abusive behaviors by caregivers are often accompanied by verbal abuse and humiliation of the victims. However, since EA represents the main predictor of ON attitudes and behaviors (Monteleone et al., 2019), it is likely that the association between PA and EA rendered the PA variable significant within the model. This implies that statistically disentangling the negative emotional experience from PA would have altered the effect of the latter on ON attitudes and behaviors – a result observable in the discrepancy between the bivariate correlation and the linear regression. The role of PA, as emerged from the regression, might reflect a differential association pattern: childhood PA appears to be more strongly linked to severe eating disorders (AN, BN, BED) than to ON. This interpretation is supported by evidence suggesting that, compared to traditional EDs, ON is associated with less impaired (Hayes et al., 2017) and more adaptive individual functioning (Rzeszutek et al., 2024). Consequently, the weaker (or paradoxical) association between PA and ON may indicate that PA predicts more severe psychopathological outcomes, while ON represents a less impairing condition along the eating disorder spectrum.

For these reasons, although considered a potential ED with significant impacts on subjective well-being, ON could be distinguished from AN, BN, and BED. These latter disorders, in addition to being recognized as more impairing EDs (Hayes et al., 2017), also tend to be more frequently associated with childhood PA (Talmon and Widom, 2022). Moreover, their strong comorbidity with Borderline Personality Disorder (Caslini et al., 2016; Miller et al., 2022), which is itself predicted by childhood PA (Salzman et al., 1993), and associated with a lower level of functioning (Kernberg, 1984) could suggest how AN, BN, and BED might be associated with less adaptive individual functioning. Thus, the findings on the weaker association between PA and ON attitudes and behaviors could support the idea that ON presents less impairing characteristics (Hayes et al., 2017), placing ON at a different level of functioning compared to AN, BN, and BED. This would be consistent with the literature’s suggestion that more impactful CT predict more severe psychopathologies (Schimmenti, 2018). Nevertheless, this interpretation ought to be approached with caution, being highly speculative and necessitating further in-depth investigation and empirical validation. Regardless, these findings raise awareness about individuals with orthorexic symptomatology and can serve as a useful clinical starting point for tailoring treatment and designing individualized therapeutic pathways.

The result concerning SA also warrants discussion. In fact, SA was excluded from the linear regression at Step 3, not being identified as a statistically significant predictor of ON attitudes and behaviors. This may appear to contradict findings from other authors regarding the role SA typically plays in predicting eating disorder symptoms (Afifi et al., 2017). However, this result aligns with the effect observed in the bivariate correlation, supporting the hypothesis that SA does not exert a statistically significant influence on ON attitudes and behaviors, possibly due to the lesser importance given to the aesthetic component in ON (Donini et al., 2022). Nevertheless, since the study involved a general population sample, the limited relevance of SA might be less pronounced compared to a clinical sample. This aspect could be explored further in future research to better understand the role of this variable on ON. In any case, this finding – supporting the notion that the aesthetic dimension is less central in ON – may be helpful for clinicians in determining what to focus on during the initial phases of patient treatment. Emotional difficulties, in fact, may require deeper intervention than, perhaps, issues related to body image distortion.

In conclusion, the final linear regression model accounted for 7.8% of the variance, showing a slight reduction compared to the initial model (8.6%). This decrease is not entirely unexpected, given that the number of predictors was reduced from five to two, and in linear regression, each independent variable contributes to the explained variance. Therefore, step-by-step elimination of variables allows for the construction of the most robust and parsimonious model possible. Moreover, a reduction in adjusted *R^2^* of less than 1% is generally considered negligible. However, it is important to note that the 7.8% explained variance is relatively modest, especially considering the significance that the literature attributes to CT in EDs (Caslini et al., 2016; Barakat et al., 2023).

However, the low proportion of variance explained in the linear regression should not be considered particularly unexpected. It is unlikely that substantial predictivity would have emerged in the analysis, given that only CT was considered as a predictor of ON. Nevertheless, by considering only CT types as independent variables, it was possible to conduct an exploratory investigation focused on their relationship with ON. As similar research has not yet been produced in the literature, this finding contributes to expanding knowledge on the role of CT on ON and lays the groundwork for more complex studies (including additional factors) in the future. In fact, the tested model deliberately excluded other potential predictors that are typically associated with ON, such as perfectionism, cognitive restraint, attachment obsessive traits, body image concerns, narcissism, and social influence. An additional possible interpretation of this modest result could lie in the limited relevance of body image within ON. In fact, according to the literature, CT tends to foster the development of a negative body image, which plays a key role in the exacerbation of EDs (Cornelissen and Tovée, 2021; Amiri and Sabzehparvar, 2025). However, as also shown in the literature, the aesthetic component in ON appears marginal and secondary to other factors, such as the need for control (Oberle et al., 2017; Cheshire et al., 2020). Thus, investigating the relationship between CT and ON may help clarify a part of its etiology that, in other EDs, tends to be more pronounced due to the centrality of the aesthetic dimension. Nevertheless, the limited number of studies examining the relationship between ON and CT suggests that these findings should not be overgeneralized, as comparable research is still scarce. Overall, this study offers a valuable contribution to both research and clinical practice, highlighting the less prominent role of CT in ON. It also suggests that future studies should further explore other aspects related to control and rigidity in ON, in connection with childhood traumatic experiences.

### Strength and limitations

The present study has some limitations that future research should take into account to further address the investigated themes. The first aspect to consider is that the model did not include certain common psychiatric comorbidities, such as anxiety or depression, which have been reported in the literature in relation to ON (Awad et al., 2021) and CT (Li et al., 2016) as well as other transdiagnostic factors related to EDs (Rossi et al., 2023a, 2023b) – such as perfectionism, asceticism of emotion dysregulation (Donini et al., 2022). Indeed, rigid behaviors in ON may serve compensatory functions, such as improving damaged self-esteem (Hymowitz et al., 2017), addressing feelings of shame and impurity (Bratman, 2017), and managing negative emotions (Pignatelli et al., 2017; Cheshire et al., 2020; Rzeszutek et al., 2024). While the present study did not directly measure these mediating mechanisms, future research should investigate whether variables such as self-esteem, shame, and emotion regulation mediate the relationship between EA and ON symptoms. Also, while their inclusion could have yielded a more comprehensive model of ON, their exclusion allowed for a more focused examination of the relationship between CT and ON, minimizing the potential confounding effects of these comorbidities. Future studies may explore the relationship among CT, ON, and comorbid psychopathologies. Another limitation of this study concerns the method of questionnaire administration: using online platforms led to the creation of a convenience sample through the snowball method. This approach may introduce selection bias, excluding individuals with certain characteristics while favouring the inclusion of others. For example, a clear underrepresentation of men was observed in the sample. However, this method is consistent with that used in many other studies on the topic (Oberle et al., 2017; Donini et al., 2022), and higher female participation in online research is considered normal (Wu et al., 2022). Therefore, in addition to the sample’s satisfactory size, the results obtained can be considered valid, and comparable with the existing literature. A second limitation to consider is the use of self-report questionnaires, which may affect data quality. The use of these instruments does not guarantee the absence of response bias, potentially related to social desirability. Moreover, in the present sample, the PN subscale of the CTQ-SF showed low internal consistency. Future studies might employ multi-method assessments, including structured or semi-structured clinical interviews, to deepen the understanding of the constructs and gather qualitative data. Alternatively, direct administration of the test battery could better control the testing conditions, reducing the presence of situational biases, and accidental or procedural errors. In any case, a data quality screening was conducted in this study, which supports the reliability of the data. Lastly, as with all cross-sectional studies, no causal relationship between the variables examined can be established. Future longitudinal studies could further explore the nature of the association between CT and ON.

Although ON is not yet formally recognized as a diagnosis with universally agreed-upon criteria and, consequently, established treatment procedures, the results presented here offer an opportunity to improve the approach to and treatment of patient cases with a condition comparable to ON. The possible presence of CT in the etiology of the condition supports its similarity with other EDs and underscores the potential benefit of therapeutic techniques that have already proven effective for them. The relevance of EA suggests that such experiences should be thoroughly explored and addressed in therapy. Moreover, the association between EA and the use of poor emotional regulation strategies points to the possible utility of EMDR (Shapiro, 2018) – that shown positive outcomes in treating patients with EDs, especially when such disorders function as maladaptive coping strategies (Morando, 2024). Otherwise, psychodynamic psychotherapy models have been developed to address childhood relational trauma: through transference and countertransference (Clarkin et al., 2006), these models allow the patient to re-experience traumatic events in a new present, restoring flexibility, and continuity to their sense of self (Bromberg, 2009).

In conclusion, the overlap that the condition of ON shares with the class of EDs – in terms of symptoms and triggering factors – seems to support treatment based on the individual’s level of functioning. The association of ON with less impaired (Hayes et al., 2017) individual functioning may allow for more positive outcomes through expressive rather than supportive therapy. Furthermore, this greater adaptability and conformity to social norms suggests that psychoeducational therapies focused on managing social, and internet influence could be effective in the treatment of ON. Nevertheless, given the still limited knowledge on ON and the absence of an official diagnosis, the proposed treatment hypotheses should be regarded with caution.

## Conclusion

Although it is a simple initial contribution of an exploratory nature, the results of this study support the association between CT and ON. EA was confirmed as the strongest predictor of ON. SA did not emerge as a statistically significant predictor, aligning with the perspective of some authors regarding the idea that the role of the body is less central in ON than in AN, BN, and BED. This suggests that clinical interventions should follow different treatment protocols. About the role of PA, further research is needed to better understand his relationship with ON, given that, based on the results, it could be hypothesized that ON could be characterized by more adaptive functioning. Additionally, experiences of neglect did not appear to be particularly relevant. This study contributes to expanding knowledge on a still underexplored aspect of ON and, consequently, to guiding psychological and psychotherapeutic interventions more effectively. Nonetheless, the result obtained from the linear regression (adjusted *R^2^* = 0.086) suggests that future studies should explore additional factors associated with ON (e.g., perfectionism, cognitive restraint, attachment, obsessive traits, depression and anxiety) and relate them to CT to gain a more comprehensive understanding of the construct. Finally, it is worth noting that this is a purely exploratory contribution, and that further research is needed – but such studies may build upon the present findings.

## Data Availability

The raw data supporting the conclusions of this article will be made available by the authors, on reasonable requests.
